# Expression of Putative Cancer Stem Cell Markers in Oral Squamous Cell Carcinoma: Correlation with Clinicopathological Features

**DOI:** 10.3390/ijms262210939

**Published:** 2025-11-12

**Authors:** Anđelija Petrović, Slavko Mojsilović, Diana Bugarski, Aleksandra Jauković, Biljana Pokimica, Miroslav P. Ilić

**Affiliations:** 1 Group for Nutritional Biochemistry and Dietology, Institute for Medical Research, National Institute of Republic of Serbia, University of Belgrade, 11000 Belgrade, Serbia; biljana.pokimica@imi.bg.ac.rs; 2 Group for Hematology and Stem Cells, Institute for Medical Research, National Institute of Republic of Serbia, University of Belgrade, 11000 Belgrade, Serbia; slavko@imi.bg.ac.rs (S.M.); dianab@imi.bg.ac.rs (D.B.); aleksandra@imi.bg.ac.rs (A.J.); 3 Center of Research Excellence in Nutrition and Metabolism, Institute for Medical Research, National Institute of Republic of Serbia, University of Belgrade, Dr Subotica 4, 11000 Belgrade, Serbia; 4 Faculty of Medicine, University of Novi Sad, 21000 Novi Sad, Serbia; miroslav.p.ilic@mf.uns.ac.rs; 5 Clinic for Maxillofacial Surgery, University Clinical Center of Vojvodina, 21000 Novi Sad, Serbia

**Keywords:** oral squamous cell carcinoma, putative cancer stem cell markers, clinicopathological features, tumor depth of invasion

## Abstract

Oral squamous cell carcinoma (OSCC) is an aggressive epithelial malignancy with high local invasiveness and a tendency for early cervical lymph node metastasis. Conventional prognostic markers often lack precision. This study evaluated the expression of putative cancer stem cell markers—CD44, CD133, and CD166—in OSCC tissues and explored their associations with clinical parameters, including salivary flow rates. Twelve patients with histologically confirmed OSCC (9 males, 3 females; mean age: 65 years) were included. Clinical TNM staging and tumor dimensions were recorded. Depth of invasion was measured histologically. Tumor tissues were enzymatically dissociated to establish primary cell cultures, and flow cytometry was used to quantify putative cancer stem cell markers expression. Unstimulated salivary flow rates were measured using sialometry. CD44 expression was uniformly high (median: 96.4%) and CD166 showed moderate to high expression (median: 85.5%), while CD133 was low (median: 1.5%). Co-expression levels were the highest for CD44+/CD166+ (median: 86.6%). Triple-marker co-expression had a median of 2.0%. Depth of invasion was positively correlated with CD133+ and its co-expression with CD44+ and CD166+ (*p* ≤ 0.05). Salivary flow rates were negatively correlated with CD166+ and CD44+/CD166+ expression (*p* ≤ 0.01). These findings suggest putative cancer stem cell markers, particularly CD133, may have prognostic value in OSCC and warrant further investigation.

## 1. Introduction

Oral carcinoma is the sixth most prevalent malignancy worldwide, exhibiting a two- to three-fold higher incidence in men compared to women [1,2,3]. Histopathological evaluation indicates that over 90% of oral malignancies are classified as oral squamous cell carcinoma (OSCC) [4,5]. OSCC represents an aggressive epithelial neoplasm characterized by varying degrees of squamous differentiation, pronounced local invasiveness, and a strong propensity for early metastasis to regional cervical lymph nodes [6]. Despite advances in treatment, the five-year survival rate remains approximately 50%, constituting a major global health concern—particularly in low- and middle-income countries, where nearly two-thirds of all new cases are reported [3,7].

One of the principal challenges in managing OSCC lies in its frequent late-stage diagnosis and high recurrence rates following treatment [8]. While histopathological analysis of biopsy specimens remains the diagnostic gold standard, there is currently no validated screening method for early detection. This diagnostic gap significantly impedes timely intervention and contributes to poor clinical outcomes. Emerging evidence highlights the pivotal role of cancer stem cells (CSCs) in tumor progression, metastasis, and resistance to therapy. CSCs comprise a subpopulation of tumor cells with the unique ability to self-renew, sustain stem-like phenotypes, and initiate tumorigenesis [9]. In addition to driving tumor initiation and clonal expansion, CSCs are implicated in therapeutic resistance and disease relapse due to their capacity for both symmetric and asymmetric division, resulting in cellular heterogeneity within the tumor microenvironment [10,11]. Despite increasing interest in CSCs, their prognostic relevance in OSCC remains an area of ongoing investigation.

In the literature, no studies were found that examined sialometry in patients with oral cancer prior to the implementation of therapeutic procedures. This parameter is most commonly assessed after radiation therapy in patients with oral cavity cancer, where xerostomia is expected as a consequence of the radiation. The unstimulated salivary flow rate ranges between 0.3 and 0.4 mL/min under physiological conditions. This rate decreases to approximately 0.1 mL/min during sleep and increases up to 4.0–5.0 mL/min during mastication [12]. According to the classification criteria for Sjögren’s syndrome, the threshold value for hyposalivation is defined as 0.1 mL/min [13].

Reliable prognostication of OSCC based solely on conventional clinical and histological parameters remains challenging. Therefore, there is a compelling need to identify sensitive and specific molecular markers that may improve early detection, prognostic assessment, and therapeutic stratification. CSC-associated biomarkers are increasingly being explored for their potential to refine prognostic models and support the development of personalized therapies. Numerous studies are focused on the detection and characterization of squamous cell carcinomas biomarkers and establishing a correlation between marker values and disease prognosis [14]. Tumor biomarkers play a crucial role in assessing disease status, predicting recurrence, and providing valuable prognostic information in oral cancer. Although various biomarkers have shown potential, further validation through well-designed clinical studies is essential for their implementation in routine diagnostics [15,16,17]. The list of potential markers for the identification of CSCs within the OSCC includes CD44, CD133, CD166, NANOG, ALDH1A1, OCT4, and SOX2 [11,18].

CD44 is a transmembrane glycoprotein involved in cell adhesion, migration, and signal transduction; it plays a critical role in tumor proliferation, metastasis, and resistance to apoptosis [19]. CD133 (prominin-1), originally identified in hematopoietic and neuroepithelial stem cells, is associated with increased tumor-initiating capacity, invasiveness, and clonogenicity in OSCC [20]. CD166, also known as activated leukocyte cell adhesion molecule (ALCAM), mediates intercellular adhesion and interaction with the extracellular matrix and is correlated with poor clinical outcomes and reduced overall survival [21].

The objective of this pilot cross-sectional study was to investigate the association between putative cancer stem cell markers expression and clinicopathological features, including sialometric profiles, in patients with OSCC. Specifically, we analyzed the expression of CD44, CD133, and CD166 in tumor-derived cell populations and assessed their correlation with unstimulated salivary flow rates and relevant clinical parameters. These findings aim to provide preliminary evidence supporting the integration of putative cancer stem cell markers into future diagnostic and prognostic frameworks for OSCC.

## 2. Results

### 2.1. Clinicopathological Characteristics of the Patients

Tumor samples from twelve patients with histopathologically confirmed OSCC were surgically excised and analyzed. All patients underwent primary tumor resection. The clinicopathological characteristics of the patients are summarized in Table 1.

Most patients were diagnosed with stage III (58.3%) or stage IV (33.3%) OSCC, while only one patient was in stage I. In 75% of patients, the depth of invasion (DOI) was <1 cm. Unstimulated salivary flow rates were within the normal physiological range in all patients, with a mean value of 0.38 mL/min.

### 2.2. Primary Tumor Cells in Culture

**Figure 1:**
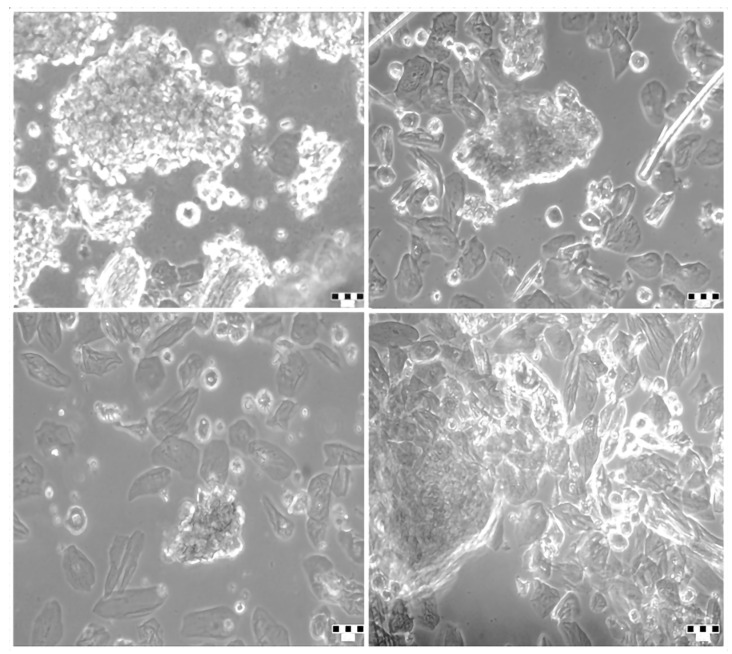
Tumor cells in culture after isolation (P0). Representative bright field microscopy images of primary tumor cells after isolation from a tumor tissue sample of an OSCC patient (patient no. 1). Scale bar: 50 μm.

To assess the prevalence of CSCs, primary OSCC cells were isolated and cultured. The adherent cells displayed characteristic epithelial morphology in vitro (Figure 1). The expression of putative cancer stem cell markers (CD44, CD133, and CD166) was evaluated using antibody staining and flow cytometry.

### 2.3. Expression of Putative Cancer Stem Cell Markers

All analyzed OSCC samples demonstrated positive expression for the putative cancer stem cell markers: CD44, CD133, and CD166.

**Figure 2:**
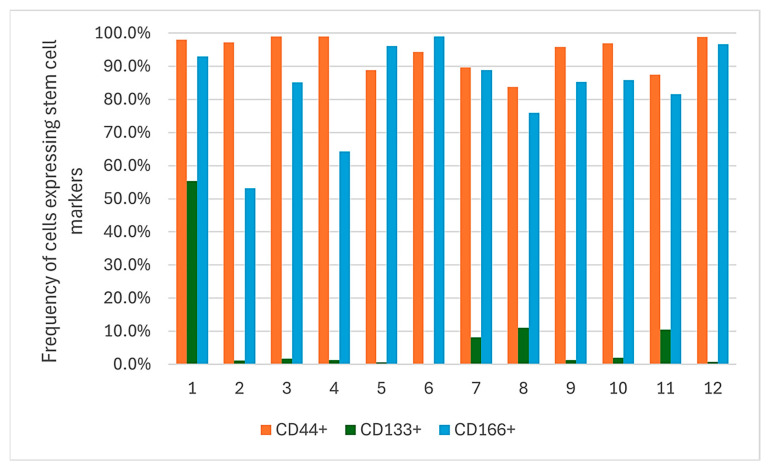
Frequency of cells expressing putative cancer stem cell markers (CD44, CD133, and CD166) in samples of oral cancer patients (n = 12).

CD44 was consistently and highly expressed across all patient samples (range: 83.8–99.0%, median: 96.4%). CD166 showed moderate-to-high expression (range: 53.1–99.0%, median: 85.5%). In contrast, CD133 expression was markedly lower, with a median of 1.5% (range: 0.3–55.4%) (Table 2, Figure 2).

**Figure 3:**
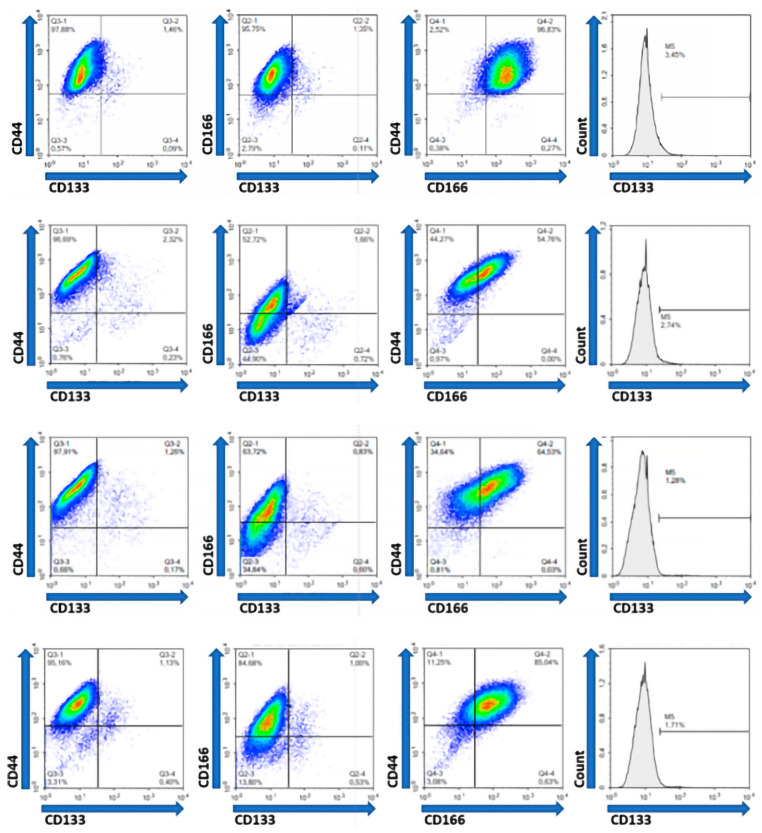
The representative results of phenotypic analysis by flow cytometry in the form of two-parameter (density) and one-parameter diagrams (histograms). The density diagrams show the simultaneous expression of the two examined markers, while the histograms show the expression of the CD133 marker within the population of cells positive for both CD44 and CD166.

Our data showed that the co-expression of CD44 and CD133, as the most frequently analyzed putative cancer stem cell markers, ranged from 0.2% to 55.2% (median 1.7%). The co-expression of CD166 and CD133 ranged from 0.19% to 53.6% (median 1.2%) and the co-expression of CD44 and CD166 ranged from 54% to 96.55% (median 86.6%) in the examined tumor tissue cell samples. The simultaneous expression of all three analyzed putative cancer stem cell markers (CD133 within CD44/CD166 double-positive population) was found to range from 0.5% to 53.6% (median 2.0%) (Table 3, Figure 3).

### 2.4. Correlation of Sialometry, Depth of Invasion, Tumor Size and Stage with Expression Putative Cancer Stem Cell Markers

To define the correlation between sialometry, depth of tumor invasion, tumor dimensions, stage of the disease and marker expression, we utilized Pearson’s correlation statistics (Table 4, Figure S1).

Our results demonstrated significant negative correlation between sialometry and the expression of CD166+ and CD44+/CD166+ (*p* ≤ 0.01). Also, there was a significant positive correlation between the depth of tumor invasion and the expression of CD133+, as well as the co-expression of CD44+/CD133+, CD166+/CD133+ and CD133+/CD166+/CD44+ (*p* ≤ 0.05).

## 3. Discussion

Compared to a quite heterogeneous tumor population, CSCs have a certain “biomarker signature” that can be determined and used for their isolation. However, since there has been no specific unique marker identified for cancer stem cells in oral and other cancers so far, several markers are usually combined for their identification [19,22]. Our study included an investigation of the cell surface markers CD44, CD133, and CD166 associated with cancer ‘stemness’, the presence of which was observed in patients with cancer of the oral cavity. In order to provide a sufficient number of cells for phenotype analysis by flow cytometry, tumor cells expanded in culture were analyzed. The phenotype analysis of cells isolated and expanded from OSCC tissue samples provided heterogeneous results for putative cancer stem cell markers (CD44, CD133 and CD166) expression. CD133 is detectable in a broad range of solid tumors, but there has been little research on its role in oral and maxillofacial tumors [23]. Our study showed that the average percentage of CD133+ cells in the analysed OSCC samples was 1.5%, which is in accordance with the results from other studies. In particular, Zhang et al. identified a small subpopulation, 1–2% of CD133+ cells in OSCC tissue [24]. Kang et al. also detected 0.95% of CD133+ cells by flow cytometry in the Tca8113 cell line (squamous cell carcinoma of the tongue) [25], while Ma et al. detected 0.41  ±  0.06% of CD133-positive cells that were isolated from primary tissue OSCC [26].

Another marker, CD44, has been associated with reduced overall survival, increased loco-regional recurrences, and increased resistance to radiotherapy in OSCC [22,27]. Our results showed that the membrane adhesive molecule CD44 was expressed on almost all tested cells from OSCC tissue samples (83.8–99.0%, median 96.4%). Data from the literature suggest that CD44 is the most commonly used marker to identify CSC from OSCC [27,28]. Ma et al. demonstrated that 33.76 ± 25.34% of primary OSCC cells express CD44 [26]. Moreover, Ludwig et al. suggested that CD44 could be the main cell marker for head and neck cancers, considering their finding on a high percentage of CD44+ cells (80–97%) in the head and neck cancer cell line TZ291013b [29]. Also, using immunohistochemical analysis, Krump et al. observed that the prevalence of CD44+ cells was significantly different in the group of patients with tongue cancer (0%) compared to the group with floor of the mouth cancer (85%) and the other groups (100%) [30].

CD166 is a leukocyte adhesive molecule, a transmembrane glycoprotein that participates in intercellular interaction and adhesion of cells to the extracellular matrix. This molecule is associated with worse prognosis and shorter survival, playing important roles in tumor initiation and propagation [31,32]. Our results showed that CD166 was expressed in a lower percentage (53.1–99.0%, median 85.5%) compared to CD44. In the study of Yan et al., the expression of CD44 and CD166 was examined in head and neck carcinoma (CAL27) cell line using flow cytometry. The incidence of CD44+ cells in this cell line was 98.68%, while the frequency of CD166+ cells reached 57.59% [33]. Also, the study by Saluja et al. showed that primary oral carcinoma cells were positive for CD44, CD133, and CD166, at various percentages. All cases showed moderate–high expressions for CD44, while the expression for other markers (CD133, CD147, and CD166) varied from mild to moderate [34]. The largest number of studies examined the expression of individual putative cancer stem cell markers in OSCC, while only a few of them examined the correlation of putative cancer stem cell markers co-expression with tumor characteristics. In our research, the co-expression of CD44 and CD133 markers, as the most frequently analyzed putative cancer stem cell markers, was found to range from 0.2% to 55.2% (median 1.7%) in cells of tested tumor tissue samples. In addition, simultaneous expression of all three analysed putative cancer stem cell markers (CD44, CD133 and CD166) was found in 0.5–53.6% of cells (median 2.0%).

In a study by Adnan et al. the expression of CD44 (33%) and CD133 (23%) was observed in OSCC patients, in addition, where the high CD44 expression was correlated with the lower survival (*p* = 0.047) while such a correlation was not identified for CD133 [35]. Our study also investigated whether there is a correlation between the expression of analysed CD44, CD133 and CD166 markers and certain clinico-pathological parameters, including sialometry, depth of tumor invasion and tumor dimensions. As for sialometry, the obtained results indicated a negative correlation showing that the expression of CD166+ alone or combined with CD44 decreased significantly (*p* ≤ 0.01) with an increase in the flow rate of saliva secretion of the investigated patients. So far, there is no literature data related to the association of the putative cancer stem cell markers expression with oral cancer patients’ salivation, particularly before the implementation of therapeutic procedures. Most often, this parameter was examined after radiation therapy of oral cancer patients, where xerostomia was expected as a consequence of radiation [36]. Considering that, a thorough study is needed on a larger number of patients to confirm the relationship between sialometry and putative cancer stem cell markers expression. In addition, our study revealed that the incidence of CD133+, CD44+/CD133+, CD166+/CD133+ and CD133+/CD166+/CD44+ cells in OSCC, increased significantly with the increase in tumor invasion depth (*p* ≤ 0.05), implying the positive correlation. Fujii et al. also found that the expression of CD133 increases significantly with an increase in the depth of tumor invasion (*p* = 0.0066), the clinical stage of the disease (*p* = 0.0424), and the appearance of regional metastases (*p* = 0.0437) [37]. In this study, the depth of invasion of OSCC ≥ 4 mm showed significantly higher expression of CD133. On the other hand, the study indicated a correlation between CD44 expression and the tumor stage, while no correlation was established with other clinicopathological characteristics. Similarly, we observed no correlation between the tumor dimensions and the assessed putative cancer stem cell markers expression using flow cytometry. However, immunohistochemical analysis conducted by Sawhney et al. showed higher expression of ALCAM (CD166) in larger OSCC (T3/T4) [38]. These discrepancies could be due to the low sample size in our study or the different method used for the marker detection. The relationship between CD44 and clinicopathological features of oral cancer still remains inconclusive. Hema et al. did not find a statistically significant relationship between CD44 expression with tumor stage, tumor localization, tumor thickness and the presence of regional metastases [39]. On the other side, high expression of CD44 was observed in the following cases: large-sized tumors, presence of regional and distant metastases, advanced TNM stage of the disease, as well as disease recurrence, all of which are associated with a poor prognosis [40]. Dhumal et al. [28] suggest that the inverse correlation between CD44 expression and prognosis in OSCC may be the result of reduced adhesion between cells, as well as between cells and the basement membrane, leading to easier detachment of cells from the rigid tissue structure. Therefore, decreased CD44 expression in OSCC tissues may serve as an indicator of high metastatic potential and be associated with lymph node metastasis and poor prognosis. Similar could also be true for CD166 expression. Indeed, Kaur et al. showed that ALCAM expression correlated with the loss of E-cadherin and β-catenin, indicating dynamic changes in the cell adhesion system in OSCC associated with poor prognosis [41]. These findings indicate that CD44, CD166, and CD133 could represent important molecular proxies for specific clinicopathological characteristics and prognostic markers of OSCC. This study contributes to the broader field of oral squamous cell carcinoma (OSCC) research by providing novel insights into the expression patterns of putative cancer stem cell markers (CD44, CD133, and CD166) in tumor tissues, and their associations with tumor invasiveness and clinical parameters such as salivary flow rates. The limitation of the present study, in terms of its design, lack of follow-ups and the small sample size, precludes the more firm conclusions of the role of these markers in monitoring treatment response and patient prognosis, so future large-scale research is warranted to corroborate our findings. Longitudinal studies with larger patient cohorts and standardized, improved methodologies are necessary to validate these preliminary observations. Additionally, employing a broader panel of biomarkers could enhance the sensitivity of early detection through molecular changes in body fluids such as saliva. Overall, further research on head and neck squamous cell carcinoma biomarkers is essential to develop precise diagnostic and therapeutic protocols tailored to molecular prognostic profiles, ultimately improving patient outcomes.

## 4. Materials and Methods

### 4.1. Study Design and Ethical Approval

This cross-sectional pilot study was conducted at the Clinic for Maxillofacial Surgery, Clinical Center of Vojvodina, and the Clinic for Dentistry of Vojvodina, Novi Sad, Serbia, between December 2018 and April 2022. The study included 12 patients with histopathologically confirmed OSCC. The cohort consisted of 9 males and 3 females, with a mean age of 65 years (range: 57–81).

Exclusion criteria included: age < 18 years; malignancies at other sites; systemic or autoimmune diseases; HIV/AIDS; acute or chronic oral inflammation; prior treatment for oral cancer; ongoing chemotherapy and/or radiotherapy; and pregnancy or lactation.

The study was approved by the Ethics Committee of the Clinical Center of Vojvodina (approval no. 00-20/654, Date 14 June 2018) and conducted in accordance with the Declaration of Helsinki. Written informed consent was obtained from all participants prior to enrollment.

### 4.2. Clinical and Histopathological Evaluation

Before surgical intervention, unstimulated whole saliva was collected using a standardized protocol. Patients were instructed to refrain from oral hygiene practices, food and drink intake, and tobacco use for at least 30 min before sample collection. Saliva was collected over 10 min using a sterile glass funnel into 15 mL tubes. We also included an assessment of salivary flow rate in our study, using a threshold value of 0.3 mL/min. Flow rates between 0.11 and 0.29 mL/min were defined as reduced salivary flow, while values ≥ 0.3 mL/min were considered within the range of normosalivation.

Tumor dimensions (D1 and D2) and clinical TNM staging (I–IV) were assessed preoperatively by the attending surgeon based on clinical evaluation [42]. Tumor depth of invasion (DOI) was determined histologically postoperatively, measured from the basement membrane to the deepest point of tumor infiltration using a calibrated eyepiece micrometer (Zeiss, Oberkochen, Germany).

### 4.3. Tumor Sample Collection and Processing

Immediately following surgical excision and prior to routine histopathological processing, tumor fragments were aseptically collected using sterile instruments.

Tissue samples were transferred into sterile 30 mL tubes containing transport medium composed of phosphate-buffered saline (PBS) supplemented with 100 IU/mL penicillin, 100 µg/mL streptomycin, 100 µg/mL gentamicin, and 2.5 µg/mL amphotericin B (Capricorn Scientific, Ebsdorfergrund, Germany). Samples were transported under sterile conditions to the Cell Culture Laboratory, Institute for Medical Research, University of Belgrade.

Under a laminar flow hood, tissues were minced into 2–3 mm^3^ fragments using sterile scalpels and anatomical forceps.

### 4.4. Enzymatic Digestion, Cell Isolation, and Primary Culture

Tissue digestion was performed using the GentleMACS™ Dissociator in conjunction with the Tumor Dissociation Kit, human (Miltenyi Biotec, Gaithersburg, MD, USA), following the manufacturer’s protocol. Briefly, 0.2–1 g of minced tissue was placed into GentleMACS C Tubes containing DMEM (Capricorn Scientific, Ebsdorfergrund, Germany) and enzyme cocktails H, R, and A.

Three mechanical/enzymatic digestion cycles were carried out using the “h_tumor_01” program, with 30 min incubations at 37 °C between cycles in an orbital shaker-incubator (ES-20, BioSan, Riga, Latvia). Following digestion, the cell suspensions were filtered through 40 µm strainers and centrifuged.

Cell pellets were resuspended in a 1:1 mixture of DMEM and Ham’s F-12 medium (Gibco, Thermo Fisher Scientific, Waltham, MA, USA), supplemented with 20% fetal bovine serum, 100 IU/mL penicillin, and 100 µg/mL streptomycin. Cell viability and counts were assessed using 0.4% Trypan blue (Gibco, Thermo Fisher Scientific, Waltham, MA, USA) exclusion under light microscopy (DM LS2, Leica, Wetzlar, Germany) with a Neubauer hemocytometer.

Cells were seeded at 1 × 10^4^ cells/cm^2^ and cultured at 37 °C in a humidified atmosphere of 5% CO_2_. The first adherent cells were designated passage 0 (P0). Medium was replaced twice weekly until subconfluence, at which point cells were washed with PBS and detached using Accutase (Capricorn Scientific, Ebsdorfergrund, Germany). Viability was reassessed using Trypan blue staining. Cells were then passaged and expanded for subsequent flow cytometric analysis.

### 4.5. Flow Cytometric Analysis of Putative Cancer Stem Cell Markers Expression

At passage 4, adherent cells were detached with Accutase (10 min, 37 °C) to preserve surface antigens. A single-cell suspension (2–5 × 10^5^ cells/tube) was prepared in PBS, centrifuged, and washed with PBS containing 0.5% bovine serum albumin (BSA; Capricorn Scientific) to block nonspecific binding.

Cells were incubated with the following primary antibodies (Table 5):

- Rabbit anti-human CD133 (ab19898, Abcam, Cambridge, UK);
- Mouse anti-human CD166-PE (BZ-343904, BioLegend, San Diego, CA, USA);
- Mouse anti-human CD44-PE-Cy7 (ab46793, Abcam, Cambridge, UK).

Following 30 min of incubation at 4 °C with CD133 antibody, cells were washed and incubated with Alexa Fluor 488-conjugated goat anti-rabbit IgG (4412S, Cell Signaling Technology, Danvers, MA, USA) for 30 min at 4 °C. Cells were then incubated with directly conjugated antibodies for CD166 and CD44 for an additional 30 min at 4 °C. After final washes, cells were fixed in 3.5% formaldehyde in PBS.

Marker expression was assessed using a FACSCalibur flow cytometer (BD Biosciences, Franklin Lakse, NJ, USA), and the data were analyzed for the presence of the specified CSC-associated surface markers.

### 4.6. Statistical Analysis

For the statistical processing of data, the commercial statistical program “SPSS 23 for Windows” was used. Data on flow cytometry are presented descriptively by means of tables and graphs, while Pearson’s correlation test was used to analyze the relationship between sialometry, tumor DOI, tumor dimensions, and marker expression.

## 5. Conclusions

In all examined primary tumor tissue samples, flow cytometry analysis demonstrated clear expression of putative cancer stem cell markers CD44, CD133, and CD166. A statistically significant correlation was observed between sialometry and the expression of CD166+ and CD44+/CD166+ populations (*p* ≤ 0.01). Previous studies have primarily examined salivary function post-radiotherapy, where xerostomia is an expected side effect. This novel approach provides new insights into the potential relationship between CSC marker expression and salivary gland function in OSCC, warranting further validation in larger cohorts.

Furthermore, there was a significant association between the depth of tumor invasion and the expression of CD133+, CD44+/CD133+, CD166+/CD133+, and the combined CD133+/CD166+/CD44+ markers (*p* ≤ 0.05). Overall, biomarkers of squamous cell carcinoma of the head and neck hold potential to facilitate the development of precise diagnostic and therapeutic protocols tailored to specific molecular prognostic profiles. This approach may pave the way for improved treatment strategies and enhanced survival outcomes in patients with head and neck cancers. While the findings of this study are noteworthy, they are insufficient to conclusively define the role and impact of tumor stem cells in the pathogenesis of oral carcinogenesis. Also, future research should investigate the biological properties of the biomarker-positive cells to confirm their characteristics as cancer stem cells, and to clarify whether these biomarkers are expressed specifically in cancer stem cells or are also present in differentiated cancer cells. Future longitudinal studies with larger patient cohorts and standardized methodologies are warranted to validate and expand upon these results.

## Figures and Tables

**Table 1:** Clinicopathological characteristics of patients with OSCC.

Patient	Stage	D1 (cm)	D2 (cm)	DOI	Sialometry (mL/min)
1	IV	3.2	2.7	2	0.3
2	III	4.5	2.9	1	0.4
3	I	2	1	1	0.3
4	III	3	2	1	1
5	IV	3	2	2	0.2
6	III	4.5	3	1	0.1
7	IV	5	3	1	0.4
8	III	4.2	3	2	0.4
9	IV	6	5	1	0.5
10	III	6	4	1	0.2
11	III	3.5	3	1	0.5
12	III	3.1	3	1	0.3

DOI—depth of invasion.

**Table 2:** Frequency of cells expressing putative cancer stem cell markers (CD44, CD133, and CD166) in oral squamous cell carcinoma samples.

Patient	Ca planocell.	CD44+	CD133+	CD166+
1	linguae	98.0	55.4	93.0
2	linguae	97.28	1.2	53.11
3	linguae	99.02	1.73	85.08
4	linguae	99.04	1.3	64.26
5	linguae	88.78	0.66	96.06
6	gingivae maxillae	94.38	0.25	99.03
7	baseos oris	89.62	8.2	88.79
8	linguae	83.81	11.02	75.95
9	linguae et baseos oris	95.89	1.36	85.24
10	linguae	96.91	1.92	85.79
11	linguae	87.41	10.49	81.61
12	linguae	98.79	0.77	96.7

**Table 3:** Frequency of cells coexpressing of putative cancer stem cell markers (CD44, CD133, and CD166) in oral squamous cell carcinoma samples.

Patient	Ca planocell.	CD44+/ CD133+	CD166+/ CD133+	CD44+/ CD166+	CD133+/ CD166+/ CD44+
1	linguae	55.2	53.6	93.1	53.6
2	linguae	1.63	0.85	54	0.51
3	linguae	1.74	1.36	86.89	2.85
4	linguae	1.18	0.8	64.51	0.73
5	linguae	0.71	1.03	87.78	0.57
6	gingivae maxillae	0.21	0.19	94.39	1.15
7	baseos oris	8.64	8.58	88.29	8.12
8	linguae	6.08	4.18	74.01	3.80
9	linguae et baseos oris	1.1	0.95	85.02	1.17
10	linguae	1.83	1.27	86.24	1.55
11	linguae	6.06	4.96	79.76	3.63
12	linguae	0.9	1.08	96.55	2.40

**Table 4:** Correlation of sialometry, depth of invasion, tumor size and stage with putative cancer stem cell markers (CD44, CD133 and CD166) expression in oral cancer.

	CD44+	CD133+	CD166+	CD44+/ CD133+	CD166+/ CD133+	CD44+/ CD166+	CD133+/ CD166+/ CD44+
Sialometry	0.113	−0.070	−0.861 **	−0.089	−0.097	−0.825 **	−0.115
DOI	−0.375	0.675 *	0.039	0.642 *	0.635 *	−0.076	0.614 *
D1	−0.153	−0.164	0.046	−0.162	−0.164	0.066	−0.175
D2	−0.082	−0.038	0.097	−0.048	−0.050	0.132	−0.060
Stage	−0.287	0.326	0.203	0.340	0.350	0.132	0.324

* Correlation significant at the 0.05 level. ** Correlation significant at the 0.01 level.

**Table 5:** Antibody combinations.

Control	Antibody
Unstained control	Alexa Fluor 488 secondary antibody only (non-specific binding and autofluorescence control)
Compensation control 1	Only CD133 and secondary Alexa Fluor 488 antibody
Compensation control 2	Only CD166-PE antibody
Compensation control 3	Only CD44-PC7 antibody
Control FMO (Fluorescence Minus One) 1	CD133, secondary Alexa Fluor 488 and CD166-PE antibodies
Control of FMO 2	CD133, secondary Alexa Fluor 488 and CD44-PC7 antibodies
Control of FMO 3	CD166-PE and CD44-PC7 antibodies
Combination of all three antibodies	CD133 with secondary Alexa Fluor 488, CD166-PE and CD44-PC7 antibodies

## Data Availability

Data are available upon reasonable request to the corresponding author.

## Supplementary Materials

The following supporting information can be downloaded at: https://www.mdpi.com/article/10.3390/ijms262210939/s1.

## Abbreviations

The following abbreviations are used in this manuscript:

ALCAM	Activated leukocyte cell adhesion molecule
CSCs	Cancer stem cells
DOI	Depth of invasion
OSCC	Oral squamous cell carcinoma
TNM	Tumor node metastasis
