# Supplementary material for: Expression of Putative Cancer Stem Cell Markers in Oral Squamous Cell Carcinoma: Correlation with Clinicopathological Features
Source: Int J Mol Sci. 2025 Nov 12;26(22):10939. doi: 10.3390/ijms262210939 (PMC12652005; doi:10.3390/ijms262210939)
Supplement: Supplementary file 1 [file ijms-26-10939-s001.zip › ijms-3865325-supplementary.pdf]

Supplementary Materials

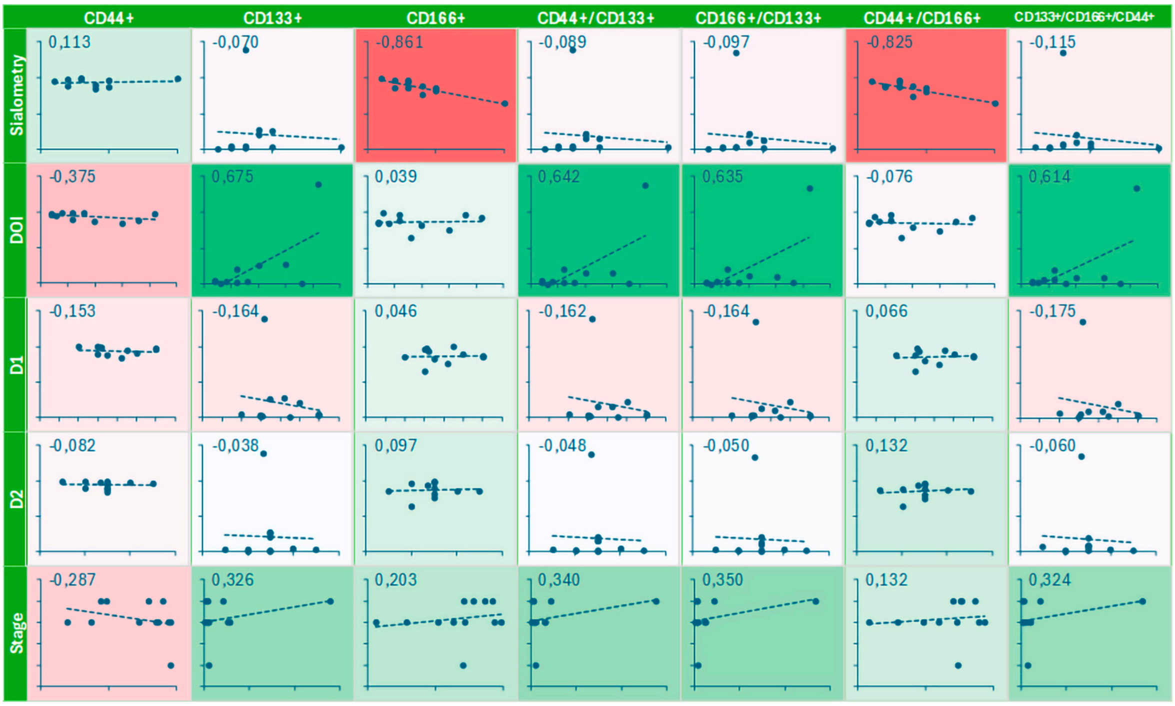

Figure S1. Correlation of sialometry, depth of invasion, tumor size and putative cancer stem cell markers (CD44, CD133 and CD166) expression in oral cancer (graphic representation of correlation). The correlation indexes are visually represented by a color-coded heatmap, ranging from red (negative correlation) to green (positive correlation). Correlations are exploratory and descriptive only.
